# Assessment of the Bending Moment Capacity of Naturally Corroded Box-Section Beams

**DOI:** 10.3390/ma14195766

**Published:** 2021-10-02

**Authors:** Przemysław Fiołek, Jacek Jakubowski

**Affiliations:** Department of Geomechanics, Civil Engineering and Geotechnics, AGH University of Science and Technology, 30-059 Krakow, Poland; pfiolek@agh.edu.pl

**Keywords:** corrosion, structural steel, hollow sections, flexural behavior, FE simulation, bending test, mechanical properties, moment resistance

## Abstract

The steel constructions of mine shaft steelwork are particularly exposed to aggressive environments, which cause large, nonuniform corrosion loss throughout the steel members. A correct assessment of corrosion loss and load-carrying capacity of shaft steelwork is crucial for its maintenance and safe operation. In this article, we present the results of laboratory, numerical, and analytical investigations conducted on naturally corroded steel guides disassembled from shaft steelwork. The steel guides considered had a closed profile formed by welding two hot-rolled channel sections. Laboratory bending tests were carried out on beams with various levels of corrosion loss, corresponding to compact, non-compact, and slender cross sections. Multiple detailed measurements of the thicknesses of naturally corroded walls were used in order to reproduce their nonuniform geometry in finite element (FE) models. The results of numerical simulations of five bending tests showed good agreement with laboratory measurements and replicated the observed failure modes, therefore confirming the applicability of this modeling approach for assessing the moment capacity of highly corroded steel beams when the deteriorated geometry is known. For the purpose of generalization, a series of derived models reflecting the natural corrosion pattern was then developed, and moment capacity statistics were collected through multiple simulations. They showed that the mean moment capacity is determined by the mean wall thickness. However, the minimum moment capacity is strongly affected by corrosion loss variation, particularly for the highly corroded beams. A simplified, analytical modeling approach was also examined, providing fairly good assessments of the mean; however, the minimum moment capacity could not be estimated. This study contributes to the body of knowledge on the mechanical behavior of highly corroded hot-rolled box-section beams.

## 1. Introduction

For the design of steel structures in aggressive environments, it is necessary to consider the impact of corrosion on the load-carrying capacity and stability of steel members. Essentially, there are two approaches to solving the problem: (1) design corrosion protection and (2) design corrosion allowances by assuming acceptable corrosion loss. One of the most important problems of structures designed with corrosion allowances is to determine the remaining load-carrying capacity of the steel members. In the literature, the load-carrying capacity assessments of corroded beams have been presented in the context of specific beam geometries, loads, and operation conditions.

Sharifi and Rahgozar [1] and Rahgozar [2] considered various forms of beam damage and highlighted the possibility of changing resistances to local buckling due to corrosion. Standard calculations for various models of corrosion loss have been used to draw the load-carrying capacity curves of corroded I-beams. The effect of corrosion on lateral torsional buckling capacity for uniformly corroded I-beam sections was investigated by Rahgozar et al. [3]. The load-carrying capacity of I-beams was expressed as a function of operating time. The curves of the torsional buckling resistance of I-beams as a function of operating time were also presented by Sharifi and Rahgozar [4]. They considered the frequently occurring [5] increased corrosion loss of the lower part of an I-beam section.

The mechanical properties of steel and the load-carrying capacity of I-beams under sulfate corrosion were investigated by Sheng et al. [6,7]. They showed the effect of corrosion in a sulfate atmosphere on the tensile failure mode of samples and reported a decrease in the load-carrying capacity of a bent beam [6]. They also showed a parabolic decrease in yield and ultimate strength with an increase in corrosion rate based on mass loss. The beam’s load-carrying capacity was reduced by approximately 5% for every 60 days in accelerated corrosion conditions. The sustained load during the exposure to the corrosive agent additionally increased the speed of the reduction in load capacity. The prediction of the ultimate load-carrying capacity of the beam under the coupling effect of the sulfate environment and extra loading using a neural network was shown [7].

Bending tests on HPS steel plate girders in three states of corrosion loss were reported by Peng et al. [8]. They analyzed the effect of chloric corrosion on decreased load-carrying capacity and its effect on the local stability of the compression flange. Laboratory tests on corroded beams combined with FE simulations have been presented [9,10,11]. Zhang et al. [9] presented the results of the laboratory bending of five beams with different corrosion loss levels achieved by outdoor artificial accelerated corrosion. They showed the effect of corrosion on load-carrying capacity in elastic and plastic states, a reduction in ductility, and an increased sensitivity to local buckling. The FEM numerical models considered uniform corrosion of the entire profile, with different material losses and pitting corrosion of individual walls. Parametric studies have been conducted in order to determine an empirical formula for estimating the load-carrying capacity of a corroded beam. Nie et al. [10] presented a study of corroded channel section beam-columns in compression and bending with respect to the weak axis. The samples came from a chemical plant building. In addition to reducing the load-carrying capacity and stability of steel members, experimental tests have suggested that corrosion has significant impacts on reducing steel strength parameters. Parametric studies have been performed by using FEM simulations, and corrosion loss has been modeled by uniformly reducing the thickness of shell elements. Wang et al. used three-dimensional (3D) scanning in order to assess corrosion loss and load-carrying capacities of beams exposed to urban atmospheric environments [11]. On the basis of laboratory and numerical tests, they determined the corrosion grade parameters and proposed an empirical formula for assessing the load-carrying capacity of beams.

The effect of corrosion on the load-carrying capacity of box girders was presented by Saad-Eldeen et al. [12,13,14,15]. To obtain corroded beam models for laboratory experiments, the samples were kept in seawater, and anodic polarization was used. Corroded box girders were used to determine the load-carrying capacity and the calibration of numerical models. The influence of local and uniform corrosion on the bending capacity of a circular hollow section has also been investigated [16,17]. Chegeni et al. [16] presented the results of pipe tests in which local corrosion was simulated by milling. Various shapes of corrosion pits (square, round, and rectangular) and their different thicknesses were tested. They showed that moment resistance was mostly determined by the depth of the pitting, as well as by a part of the diameter of the dangerous cross section it covered, while the shape of the corrosion pitting itself was negligible. Elchalakani [17] investigated the effect of the uniform corrosion of CHS on load-carrying capacity. Five corrosion loss levels (from 0 to 80%) were simulated by turning. The determined experimental load-carrying capacity was significantly lower than the bending moment resistance calculated on the basis of standard calculations due to the significant influence of shear force in the three-point bending test. The effect of uniform corrosion on channel sections welded to form a box-section beam was investigated by Fiołek et al. [18] and Fiołek and Jakubowski [19]. By performing laboratory and numerical bending tests, they showed the effect of uniform corrosion on the local buckling resistance of analyzed box beam profiles. To produce the high corrosion loss, they used etching with hydrochloric acid. In particular, they showed that, in mine shaft conditions, a significant profile cross-section reduction occurred, and hot-rolled profiles could be sensitive to local buckling in an elastic state.

The flexural behaviors of corroded I-sections [1,2,3,4,5,6,7,8,9,11], U-channels [10], plated structures [12,13,14,15], and circular hollow sections [16,17] are well described in the literature, but there is a lack of research studies performed on corroded, rectangular hollow sections. The uniform corrosion of box-section beams has been previously examined [19]. In this study, the aim is to analyze the influence of nonuniform, natural corrosion on the load-carrying capacity of rectangular hollow section beams made of hot-rolled channels. These profiles are used as guides in the construction of shaft steelwork [20] and are exposed to atmosphere [21,22], erosion [23], and mine water [24] corrosion. Furthermore, high humidity and temperature resulted in high corrosion loss, which is unusual for civil engineering structures [18,19,25]. The tested beams were exposed to an underground corrosion environment for over 30 years. In order to consider naturally occurring nonuniform corrosion loss, bending tests were carried out on five corroded sections of the steel guides obtained from a deep coal mine shaft. Detailed geometry measurements were performed and reflected using FE model geometry. Elastic and ultimate moment capacities were investigated by laboratory tests, FE simulations, and analytical formulas.

## 2. Specimens, Tests, and Models

### 2.1. Specimens and Material Properties

**Figure 1:**
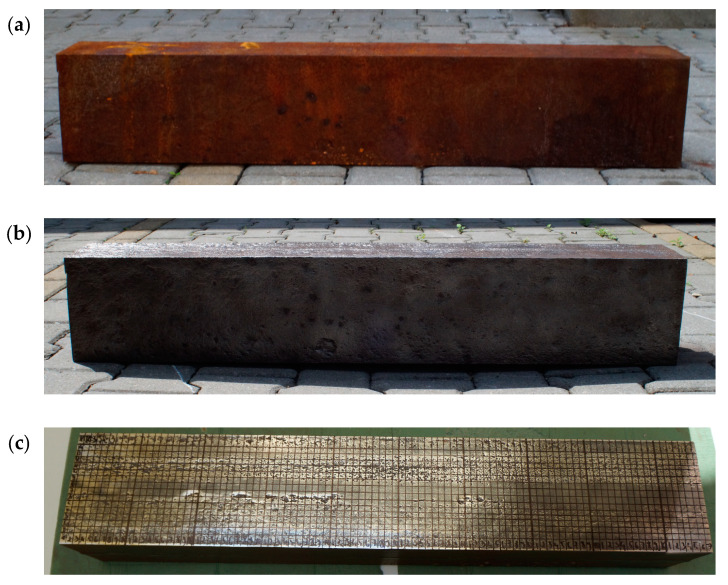
Guide section taken from the shaft steelwork: (**a**) before; (**b**) after cleaning; (**c**) mesh view on web.

**Figure 2:**
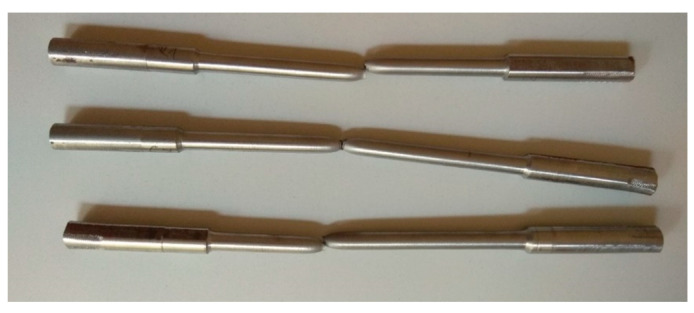
Failure mode of the tensile samples.

The tests were performed on shaft guide segments that were disassembled in a coal mine shaft as part of cyclic renovation due to significant corrosion loss (Figure 1). The guides are welded with two C180 hot-rolled channels [19]. In pursuance of shaft technical documentation, the guides are made of 18G2Cu steel according to the standard [26], which corresponds to steel grade S355 according to [27]. Tensile tests were carried out to determine yield strength and ultimate strength. Tests of the strength parameters of the material of shaft steelwork members taken from the mine shaft were performed on round specimens. The samples were taken randomly from the channel flanges. The samples were then machined to obtain standard proportional test pieces [28]. Uniaxial tension tests were carried out on six specimens. Tests on round specimens allowed capturing the deterioration of the mechanical properties of the material caused by intergranular corrosion, stress corrosion, or sulfide stress cracking [29,30] and avoided the geometric notches [6,31,32]. The mechanical properties obtained on round specimens adequately characterized the material properties, while the geometry of the corrosion pattern on the surface is represented by nonuniform shell element thickness distribution. The test results are presented in Table 1. The average yield strength (σ_y_) and ultimate strength (σ_u_) were 390 and 600 MPa, respectively. All specimens showed typical ductile fractures (Figure 2). The yield strength and ultimate strength both reached their respective average values for steel S355 [33,34]; the reductions in steel strength properties due to corrosion reported in the literature [10,31,32,35] were not detected.

**Figure 3:**
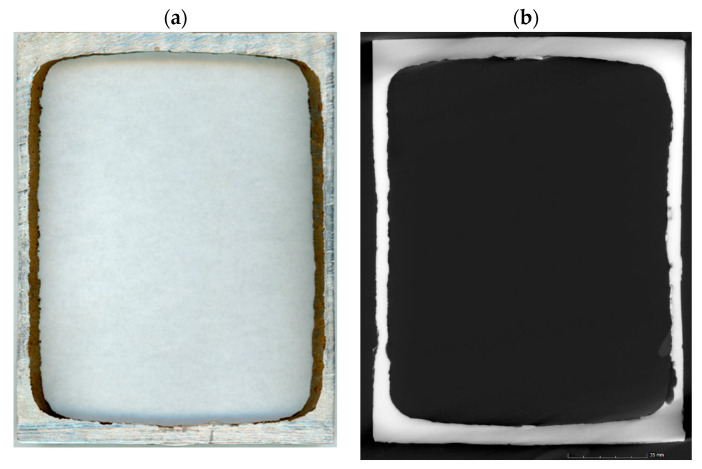
Guide cross section (**a**) and CT scan (**b**).

Random surface and crosscut inspections and computer X-ray tomographic (CT) scans of the guide cross section (Figure 3) did not show the microcracks in the channel walls. Additionally, the quality of welds connecting two channel profiles was verified. The welds showed full penetration [36].

**Figure 4:**
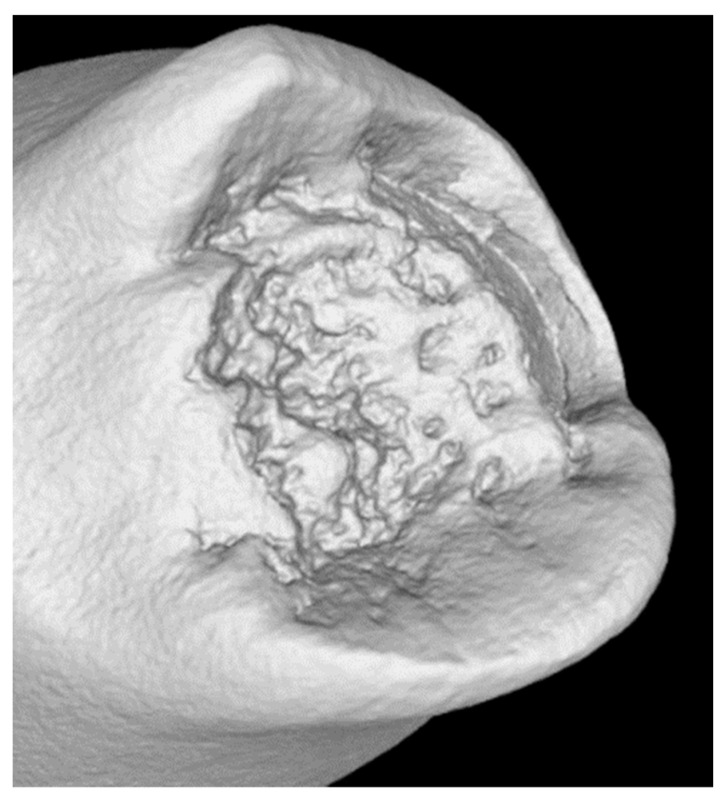
Sample after tensile test CT reconstruction.

The X-ray CT tomographic examinations with phoenix v|tome|x m equipment (GE, Boston, MA, United States) also confirmed that corrosion did not affect the failure mode of the tested specimens under uniaxial tension (Figure 4). The crack area did not indicate local weakening of the material causing a brittle fracture.

### 2.2. Bending Test Setup

**Figure 5:**
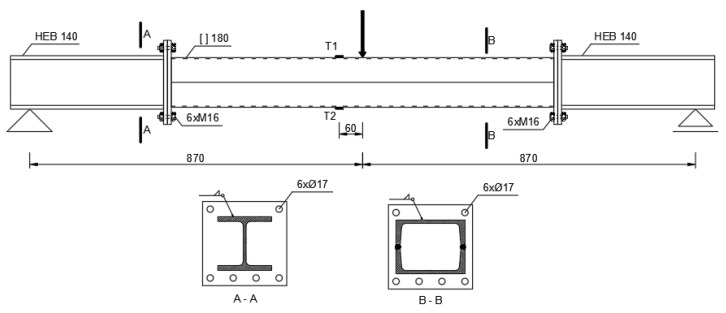
Diagram of the experimental setup (dimensions in millimeters).

A series of three-point bending tests were applied to the beams. During bending, the strain on the upper (T1) and lower (T2) wall was measured using strain gauges. In order to reduce the effect of transverse force, the lengths of the samples were extended by using two HEB 140 H-section segments installed on both sides of the box sections. The stiff H-beam extensions did not interfere with the results. The test diagram of the experimental setup is shown in Figure 5. The beams were bent at a load rate from 1 kN/s to destruction.

### 2.3. FE Model

**Figure 6:**
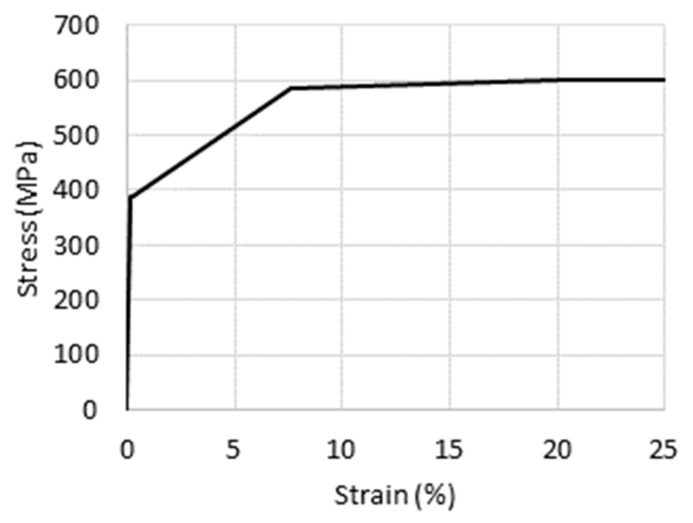
Stress–strain curve of the applied material model.

The simulations of the three-point bending test were carried out in Abaqus 6.12 [37]. The connection between box-section beams and extension elements was made by using the “tie” constraint [37]. The quad-linear hardening material model [38] is based on the maximum shear strain energy (von Mises or Huber) yield criterion. The stress–strain curve of the applied material model is shown in Figure 6. The Young’s modulus and Poisson’s ratio of the box section and extension elements were set to 210 GPa and 0.3, respectively [39].

**Figure 7:**
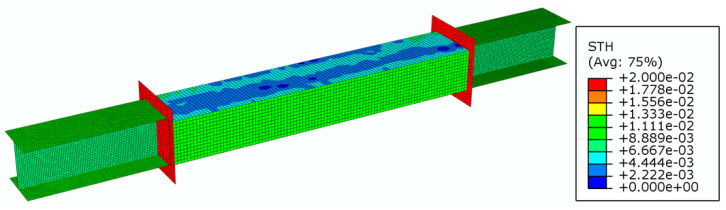
Shell element thickness (STH) map for a sample beam section (m).

Due to the expected bending deformation and high strain gradient (local buckling), the quad four-node shell elements with reduced integration (S4R) [37] were applied. The wall thickness pattern was represented by the nonhomogeneous thickness of the finite element mesh, according to the ultrasonic measurements of the guide sections. Preliminary analysis of the impact of the finite element mesh size on the simulation results was carried out. Figure 7 shows the model of one of the specimens. The extension elements were mapped with S4R elements of a uniform thickness. A geometrically and materially nonlinear numerical analysis was carried out by using the Abaqus/Standard module and the Riks method [40].

### 2.4. Simplified Corroded Beam Geometry and Analytical Model

Numerical simulations of load-carrying capacity with models reflecting nonuniform corrosion loss could be considered too demanding for routine practical applications. Therefore, it is attractive to represent a nonuniform thickness geometry of a real corroded guide with an averaged, uniform wall thickness geometry. The results of such an approach were investigated in this study and compared with other corresponding results.

The question is how to determine the simplified geometry assembling uniform thickness walls for a nonuniformly corroded profile. By using the thickness measurements (Figure 2), the volume of a 30 cm long wall at the center of the beam can be calculated and then divided by the area in order to find the averaged thickness of the considered wall according to Equations (1)–(4).

(1) $$t_{\mathrm{wu}}=\frac{\mathrm{upper}\;\mathrm{web}\;\mathrm{volume}}{\mathrm{upper}\;\mathrm{web}\;\mathrm{area}}$$

(2) $$t_{\mathrm{wb}}=\frac{\mathrm{bottom}\;\mathrm{web}\;\mathrm{volume}}{\mathrm{bottom}\;\mathrm{web}\;\mathrm{area}}$$

(3) $$t_{f1}=\frac{\mathrm{first}\;\mathrm{flange}\;\mathrm{volume}}{\mathrm{first}\;\mathrm{flange}\;\mathrm{area}}$$

(4) $$t_{f2}=\frac{\mathrm{second}\;\mathrm{flange}\;\mathrm{volume}}{\mathrm{second}\;\mathrm{flange}\;\mathrm{area}}$$

**Figure 8:**
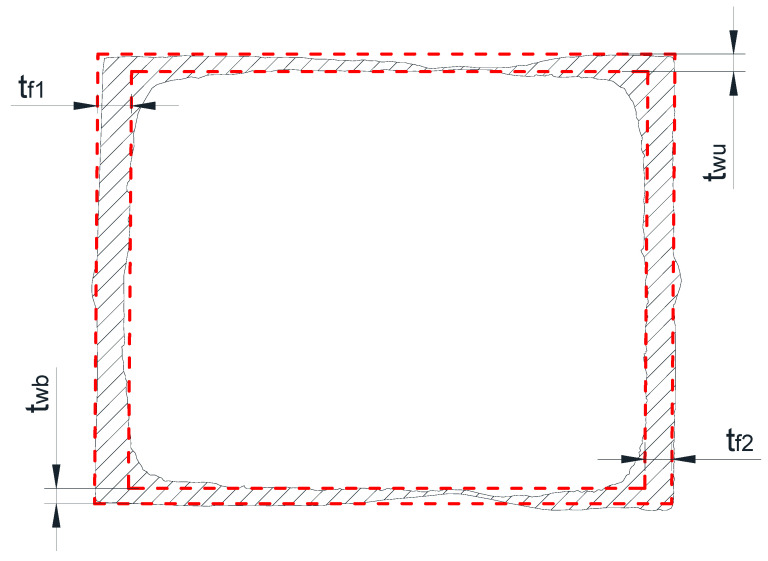
Illustration of the simplified cross-sectional geometry.

The cross-sectional depth and width are assumed to be nominal. The shape of the simplified section geometry with a uniform wall thickness is shown in Figure 8. The measurement results are presented in Table 2. The elastic (W_el_) and plastic (W_pl_) section moduli were also calculated.

By representing the corroded beam walls using a simplified, uniform thickness geometry, it is possible to assess the load-carrying capacity with respective analytical formulas. The elastic (M_R,el_) and plastic (M_R_) bending resistance about the weak axis is given by Equations (5) and (6), respectively.

(5) $$M_{R,\mathrm{el}}=W_{\mathrm{el}}\sigma_{y}$$

(6) $$M_{R}=W_{\mathrm{pl}}\sigma_{y}$$

On the basis of the section modulus (Table 2) and the yield strength determined in the tensile tests (390 MPa), the elastic and plastic bending moment resistance was calculated according to Equations (5) and (6). The results are presented in Section 3.5.

## 3. Results and Discussion

### 3.1. Corrosion Loss Distribution Measurements

**Figure 9:**
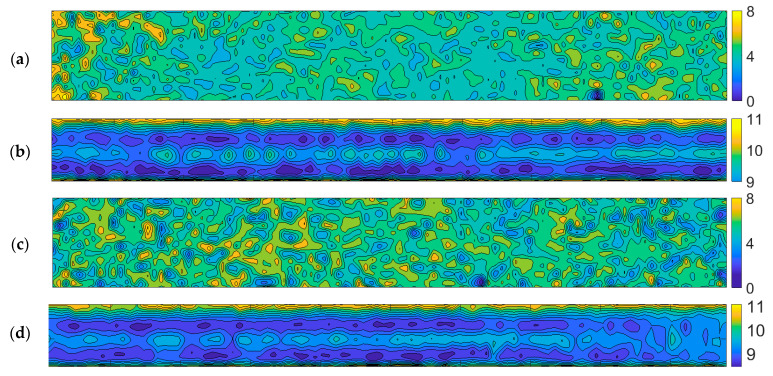
Maps of web (**a**,**c**) and flange (**b**,**d**) thickness distributions (mm).

In order to reproduce the corrosion pattern, thickness measurements were performed in a dense, regular mesh of points. The specimens were cleared of rust by electric wire brushes and marked with a 1 cm × 1 cm mesh (Figure 1c). The thickness measurements were performed with a PosiTector ultrasonic thickness gauge (DeFelsko, Ogdensburg, NY, USA), and they were used to produce maps of the web and flange thickness distributions. Sample maps are presented in Figure 9.

### 3.2. Laboratory Bending Tests and Their FE Simulations

**Figure 10:**
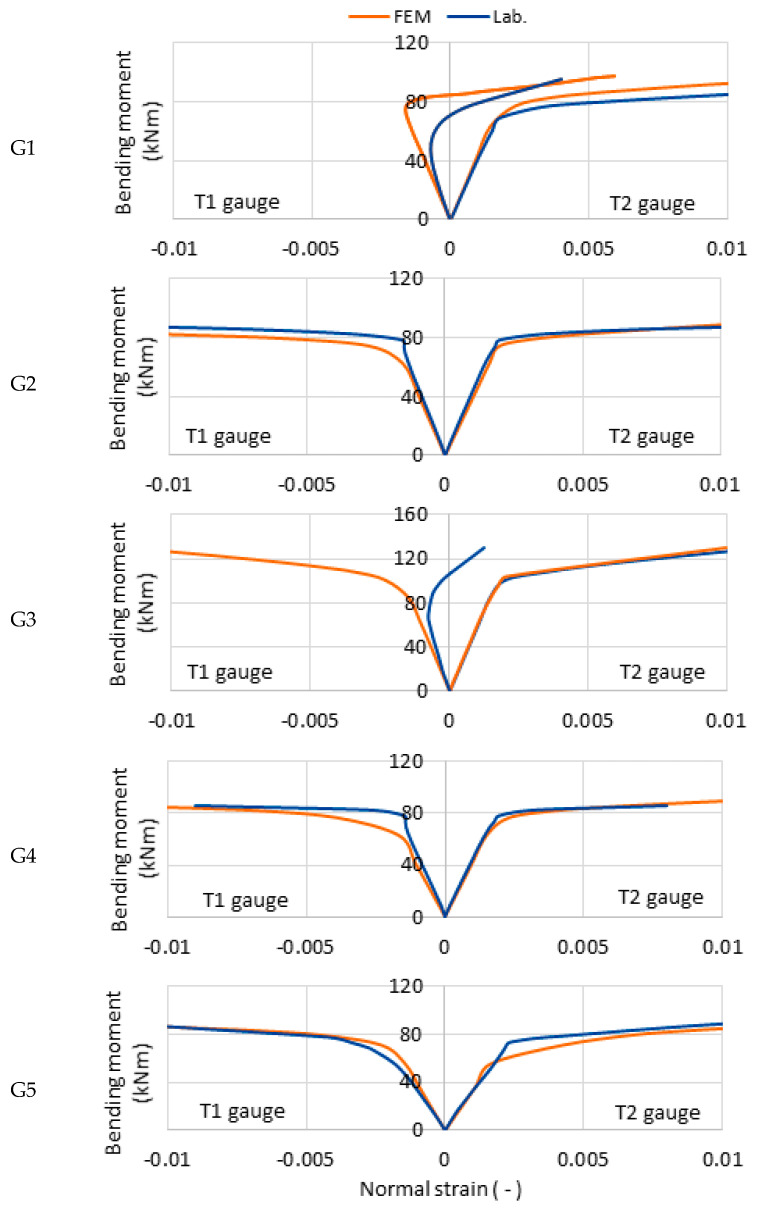
Moment–strain relationship diagram obtained from the laboratory tests and numerical simulations of five naturally corroded beams (**G1**–**G5**). The strain readings at the top wall (under compression, T1) are shown on the left, whereas the strain reading at the bottom wall (under tension, T2) are shown on the right.

The five guide segments were tested in a three-point bending test, as described in Section 2.3. According to the guide thickness measurements, five respective FE models representing nonuniform corrosion patterns of the original beams were built, as described in Section 2.2. The normal strains measured during the laboratory experiments and evaluated by the numerical simulation were compared. Figure 10 shows the strain readings at T1 on the upper wall of the profile under compression (plots directing left from the origin) and at T2 on the lower wall of the profile under tension (plots directing right from the origin). The results of the numerical simulations are in good agreement with the test results.

The load-carrying capacity of the G1 beam simulation reached 95.7 kNm. For a bending moment greater than 70 kNm, the normal strain values increased rapidly, indicating the occurrence of plastic deformation, and the beam, therefore, achieves plastic moment resistance. The load-carrying capacity of the G2 beam simulation reached 87.9 kNm; for moment values less than 80 kNm, the moment–strain characteristics behaved linearly; and the range of plastic load-carrying capacity is small compared with the G1 beam simulation. The ultimate moment capacity of the G3 beam simulation reached 130.5 kNm; the moment–strain diagram is similar to the G1 beam simulation’s diagram; and the strain diagram on a compressed wall loses linearity at lower bending moment values, which is typical for three-point bending. The load-carrying capacity of the G4 beam simulation reached 86.3 kNm; curves describing beam deformations retained their linear nature nearly until destruction; the absence of large plastic deformation indicates a lack of plastic moment resistance; and the effects of local buckling were observed on the upper surface. The load-carrying capacity of the G5 beam simulation reached 106.1 kNm; for a moment of 70 kNm, the deformation curves are linear; the high ultimate moment capacity is surprising given the perforations in the web due to corrosion; and local buckling was observed on the upper surface.

**Figure 11:**
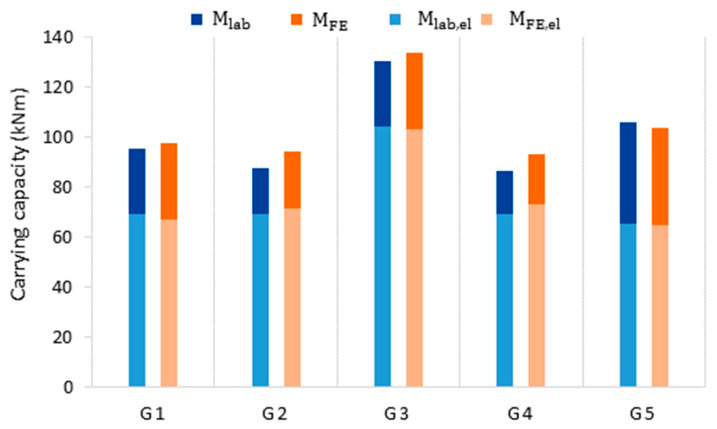
Elastic and ultimate moment capacity obtained from laboratory test (**blue**) and numerical simulations (**orange**).

The moment–strain curves achieved from the laboratory tests and numerical simulations show very good agreement. The moment–strain curves on the compression wall show greater nonlinearity related to the loss of local stability due to compressive stresses and the effect of force application. This behavior is typical [19], and it is more visible and reflected by the laboratory tests results. High agreement observed for the plastic state (particularly tensile wall) indicates the correct selection of the material model. The strain measurement allows one to determine the transition of the profile from the elastic to the plastic state. A summary of the elastic and ultimate moment capacities obtained from laboratory tests (M_lab,el_ and M_lab_) and numerical simulations (M_FE,el_ and M_FE_) is presented in Table 3. The differences between the elastic moment capacity, calculated in the simulations and measured during the laboratory tests, range from 0.5% to 5.6%. The material transition to a plastic state captured during laboratory and numerical experiments largely depends on the web thickness under the strain gauge and the corresponding finite element thickness, respectively. Thus, the captured yield is subject to high variations resulting from random thickness distribution. The differences between the ultimate moment capacity, calculated from FEM simulations and that obtained from laboratory tests, range from 1.8% to 8.2%. The results of laboratory tests and numerical simulations are presented in Figure 11.

The G5 sample with the highest deterioration of the webs (perforations) achieved a load-carrying capacity greater than the seemingly less corroded samples (G1, G2, and G4) due to its slightly corroded, heavier flanges.

**Figure 12:**
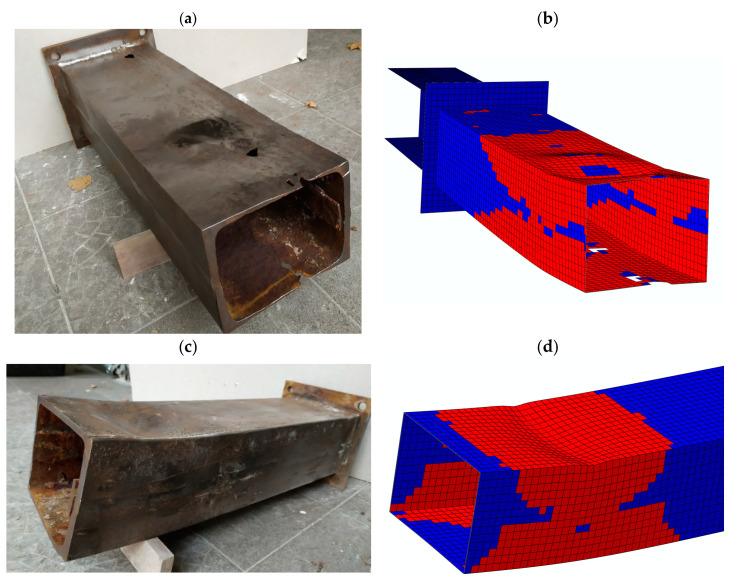
Failure mode views after destruction: (**a**) G5 sample; (**c**) G3 sample. Distribution of the yield elements at the maximum bending moment: (**b**) G5 bending simulation; (**d**) G3 bending simulation.

The numerical simulations also effectively reproduced the respective failure modes. The G5 sample, with the thinnest web, showed the form of failure characteristics for a slender cross section (four class by [39]) and a clear buckling wave (Figure 12a,b). The G3 guide sample of insignificant corrosion loss showed the form of failure characteristics for the compact (one or two class by [39]) cross sections (Figure 12c,d). The length of the developing plastic hinges reached about 30 cm.

### 3.3. The Influence of the Central Segment Length

**Figure 13:**
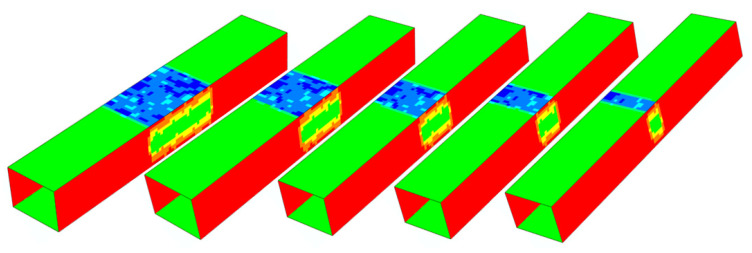
Model view illustrating the segment length effect investigations. Shell element thickness maps are shown.

The applied three-point bending load diagram implies that the maximum bending moment, the ultimate moment, and the plastic hinge developed in the central segment of the beam; therefore, the central segment determines the load-carrying capacity of the beam. The question is, “What is the significant length of the central segment?” Specifically, “Is the 30 cm segment sufficiently long to avoid its length’s impact on moment capacity results?” In order to answer this question, two preliminary simulation series were carried out on the G1 and G2 sample beam models for eight central segment lengths ranging from 2 to 42 cm. The central segments of the models were copied from the original naturally corroded beams. For the remaining parts of the beams, nominal values of wall thickness were adopted (Figure 13).

**Figure 14:**
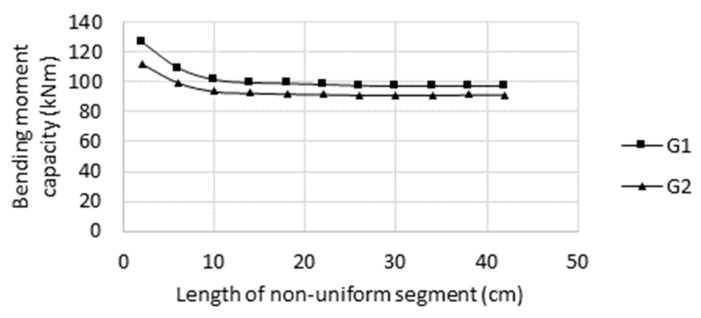
Impact of naturally corroded segment length on the simulation results.

The effect of the naturally corroded segment length on the ultimate moment capacity is shown in Figure 14. For segment lengths greater than 10 cm, the decrease in load-carrying capacity along with the segment length is small. For segment lengths greater than 30 cm, no decrease is observed, which indicates that the 30 cm central part is long enough for determining the moment capacity of the beam accurately and to avoid result bias due to the limited length. This observation is employed later for the investigations presented in Section 3.

### 3.4. Derived FE Models Representing Natural Corrosion Patterns

The models and simulations presented in Section 2.3 and Section 3.2 were used to evaluate the moment capacity statistics of the five investigated guide sections, representing the geometry of the five sections tested in the laboratory. However, the moment capacity was determined by the limited 30 cm long central section of the beam. The available measurements of beam thickness along its full length provided information on the corrosion in the central segment and through the full length of the original guide segment, which was previously unused. This information was used to produce 28 derived geometries based on 30 cm segments of the original beam. These 28 models for each of the five original beams resulted in 140 geometric models, representing natural corrosion patterns of the five original beams. The simulations with these multiple models reflected the moment capacity statistics and extended the results from the previous section, with mean values and dispersion estimates. This approach extended the range of results, the accuracy of the mean value assessment, and allowed for the quantification of this accuracy.

**Figure 15:**
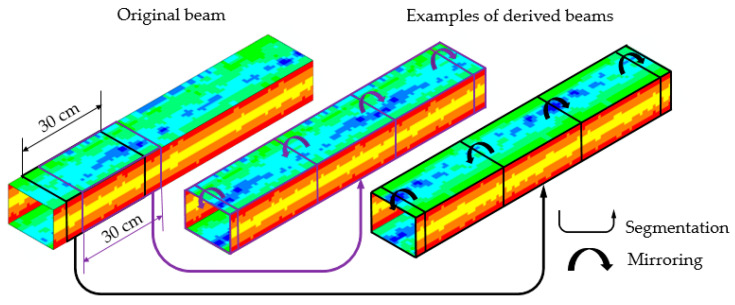
Shell element thickness for beams created by original beam segmentation and then by mirroring the segments. The color (from blue to red) indicates element thickness.

Multiple numerical simulations were conducted on the models based on 30 cm segments of an original beam and forming the central part of a newly derived beam. The remaining parts of the derived beam were completed by mirroring the central part towards both ends of the beam to provide smooth transitions along the beam length and to prevent unnatural edges. Figure 15 shows a few beams created by segmentation and mirroring. The color (from blue to red) indicates element thickness.

As explained above, for each of the five guide geometries, 28 derived models were built. The continuity and natural variability of corrosion loss of the original beam was preserved and reflected by models. These models were used to investigate the impact of a nonuniform, natural corrosion pattern and its variation on load-carrying capacities.

**Figure 16:**
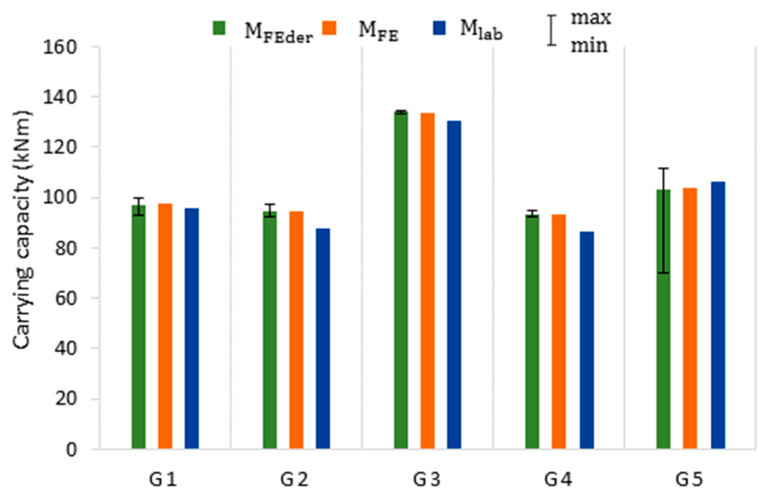
Ultimate moment capacity of laboratory (**blue**) and numerical (**orange**) experiments on original beams and numerical experiments on derived beams (**green**). The whiskers show maximum and minimum moment capacities for derived beams.

**Figure 17:**
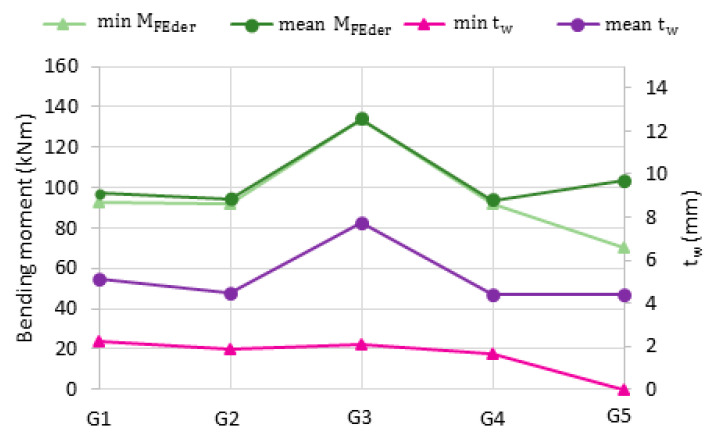
Minimum and mean values of web thickness (**purple**) and moment capacity (**green**) for derived beams samples.

Table 4 describes the results of bending simulations on 140 models (28 for each beam) created by sectioning and mirroring the original beams (G1–G5). The statistics of the load-carrying capacity (M_FEder_) and web thickness (t_w_) were presented. The results are also shown in Figure 16 and Figure 17.

**Figure 18:**
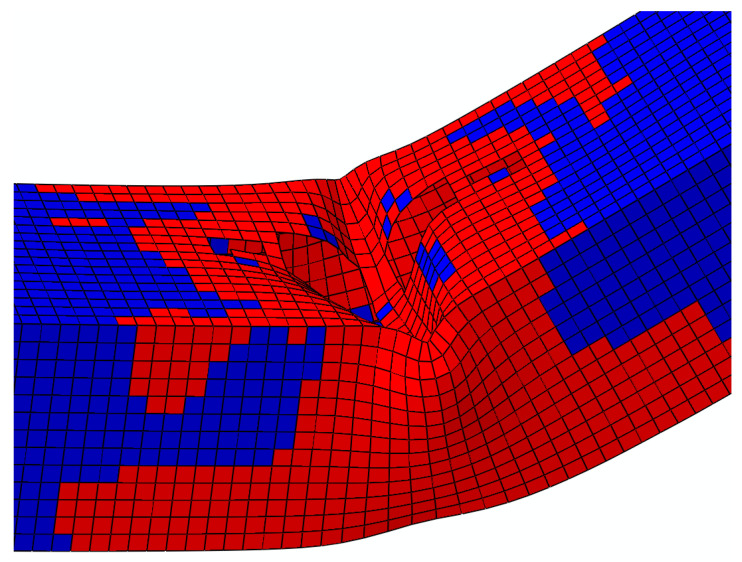
The failure mode of the G5-derived beam showing the minimum moment capacity.

The highest thickness coefficient of variation is observed for the G5 guide, in which corrosion resulted in the formation of local web perforations in the compression wall. The highest ultimate moment capacity coefficient of variation is also observed for models built from the G5 guide. For the others beams, the coefficients of variation in moment capacity are very low (0.23–1.83%), indicating that their corrosion pattern variation does not convert to moment capacity variation. The mean web thickness (and not the nonuniform corrosion pattern variation) seems to impact the mean moment capacity of all the beams, including the G5 beam corroded with perforations. The minimum load-carrying capacities of the moderately corroded G1–G4 beams differed slightly from their respective mean values. On the other hand, for the perforated G5 beam, the minimum moment capacity (Figure 16) is significantly lower than its mean value. Therefore, the nonuniform corrosion pattern variation strongly impacts the minimum moment capacity of the locally perforated G5 beam (Figure 18).

### 3.5. Analytical Model

Numerical simulations of load-carrying capacity with models reflecting nonuniform corrosion loss might be considered to be too complex and exhausting for routine practical applications. The solution investigated here is to represent nonuniform thickness geometry of the real corroded guide with averaged uniform wall thickness geometry, as described in Section 2.4. Such simplified geometry can be used to perform load-carrying capacity assessments according to known analytical formulas (Equations (5) and (6)).

**Figure 19:**
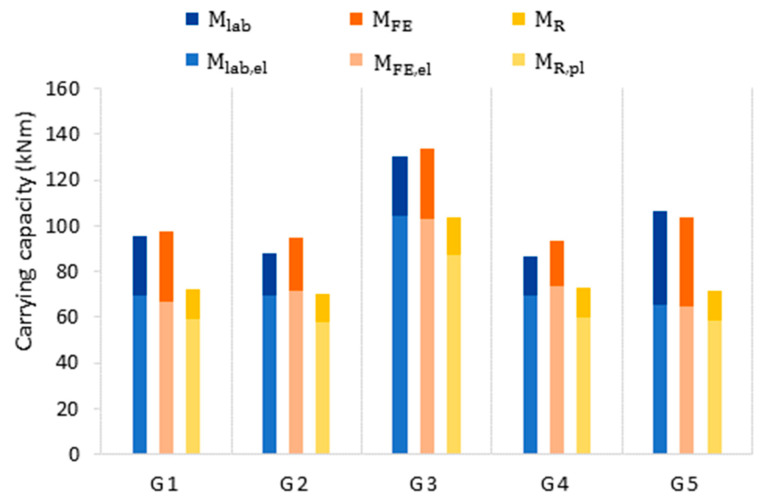
Comparison of the load-carrying capacity assessment in laboratory tests (**blue**), FE models of the original beams (**orange**), and analytical formulas (**yellow**).

For the five original beams, the averaged uniform wall thicknesses and section moduli, i.e., elastic (M_R,el_) and plastic (M_R_) bending moment resistance, were calculated (Table 5). The elastic and plastic moment resistances were then assessed by Equations (5) and (6). The results are presented in Table 5 and in Figure 19.

Table 5 presents the results of analytical calculations. Analytical calculation results were compared with the corresponding results of laboratory tests (M_lab,el_ and M_lab_), as shown in Figure 19.

The moment resistance of the cross section, calculated from by using analytical formulas, is significantly lower than the moment capacity obtained in laboratory tests. The elastic moment resistance M_R,el_ is up to 16.7% lower, and the plastic moment resistance M_R_ is up to 32.8% lower than the respective test moment capacities. The analytical assessments are on the safe side and can be considered for quick design methods; however, they cannot be used for quantitative reliability assessments.

## 4. Conclusions

In this article, we presented the results of a bending moment capacity assessment of naturally corroded box-section beams made of hot-rolled channels. These sections are widely used in the mining industry as guides for shaft conveyance and are exposed to high corrosion loss. The load-carrying capacity of corroded beams was determined on the basis of laboratory tests, numerical simulations, and analytical formulas.

(1) Corrosion processes that caused a significant local reduction in the cross-sectional area and perforation did not cause a noticeable reduction in the strength properties of steel material. The tensile tests were carried out on round pieces drawn from the thickest part of the profile. Therefore, they did not have notches or other stress concentrations reducing the yield strength, ultimate strength, and elongation.
(2) The moment resistances obtained on the basis of the analytical formulas are lower than the values obtained by laboratory tests and numerical simulations. This refers not only to the ultimate moment capacity, which could be explained by the lack of consideration of steel hardening, but also to elastic moment capacity.
(3) The comparison of the results obtained from laboratory tests and the respective numerical simulations indicates high agreement in the results. Moreover, the numerical simulations effectively reproduced the respective failure modes and local buckling resistance for compact (G3), non-compact (G1, G2, and G4), and slender (G5) cross sections.
(4) Numerical simulations on derived models allow for drawing conclusions based on 28 element samples reflecting the natural corrosion pattern of each of the G1–G5 beams. The minimum moment capacity is strongly affected by corrosion loss variation for the highly corroded beam with perforations (G5). It is worth mentioning that this conclusion could not be drawn without multiple numerical simulations conducted on derived models reflecting the natural corrosion pattern. This approach can be used to investigate the variability of moment capacities resulting from the natural corrosion pattern, which appeared to be negligible for the G1–G4 beams but significant for the G5 beam.

## Figures and Tables

**Table 1:** Results of the tensile tests.

Sample	σ_y_ (MPa)	σ_u_ (Mpa)
1	395.2	608.1
2	398.3	606.2
3	392.3	597.3
4	389.5	609.9
5	395.0	600.9
6	382.5	612.1

**Table 2:** Geometric characteristics of the simplified geometry.

Beam	t_wu_ (mm)	t_wb_ (mm)	t_f1_ (mm)	t_f2_ (mm)	W_el_ (cm^3^)	W_pl_ (cm^3^)
G1	4.53	5.72	9.01	9.28	151.9	184.7
G2	4.14	4.79	8.81	7.55	149.1	179.1
G3	7.71	7.87	10.37	10.40	222.9	265.7
G4	4.34	4.42	9.11	9.27	153.0	186.0
G5	4.18	4.21	9.25	9.29	149.7	182.7

**Table 3:** Comparison of the load-carrying capacities obtained from laboratory tests and FEM simulations.

Beam	Laboratory Test		FE Simulation		Relative Difference	
	Ultimate M_lab_ (kNm)	Elastic M_lab,el_ (kNm)	Ultimate M_FE_ (kNm)	Elastic M_FE,el_ (kNm)	Ultimate Capacity (%)	Elastic Capacity (%)
G1	95.7	69.6	97.4	66.9	1.8	−3.9
G2	87.9	69.3	94.6	71.3	7.6	2.9
G3	130.5	104.4	133.9	103.2	2.6	−1.1
G4	86.3	69.6	93.4	73.5	8.2	5.6
G5	106.1	65.3	103.8	64.9	−2.2	−0.5

**Table 4:** Statistics of ultimate moment capacity and web thicknesses of beams derived by original guide segmentation and mirroring.

Original Guide	M_FEder_				t_w_			
	Min (kNm)	Max (kNm)	Mean (kNm)	CoV (%)	Min (mm)	Max (mm)	Mean (mm)	CoV (%)
G1	92.7	99.9	97.0	1.83	2.25	8.8	5.13	20.3
G2	92.1	97.5	94.3	1.28	1.88	8.49	4.45	18.4
G3	133.5	134. 8	134.0	0.23	2.05	8.87	7.78	6.9
G4	92.2	95.0	93.5	0.76	1.62	7.54	4.41	22.9
G5	70.3	111.4	103.5	8.11	0	8.14	4.37	34.9

**Table 5:** Comparison of the load-carrying capacities obtained from laboratory tests analytical model.

Beam	Laboratory Test		FE Simulation		Relative Difference	
	Ultimate M_lab_ (kNm)	Elastic M_lab,el_ (kNm)	Ultimate M_R_ (kNm)	Elastic M_R,el_ (kNm)	Ultimate Capacity (%)	Elastic Capacity (%)
G1	95.7	69.6	72.0	59.2	−14.9	−24.7
G2	87.9	69.3	69.9	58.2	−16.1	−20.5
G3	130.5	104.4	103.6	86.9	−16.7	−20.6
G4	86.3	69.6	72.6	59.7	−14.3	−15.9
G5	106.1	65.3	71.3	58.4	−10.5	−32.8

## Data Availability

The data presented in this study are available on request from the corresponding author.
